# Supplementary figures and images for: BRG1 HSA domain interactions with BCL7 proteins are critical for remodeling and gene expression
Source: Life Sci Alliance. 2023 Feb 17;6(5):e202201770. doi: 10.26508/lsa.202201770 (PMC9939006; doi:10.26508/lsa.202201770)

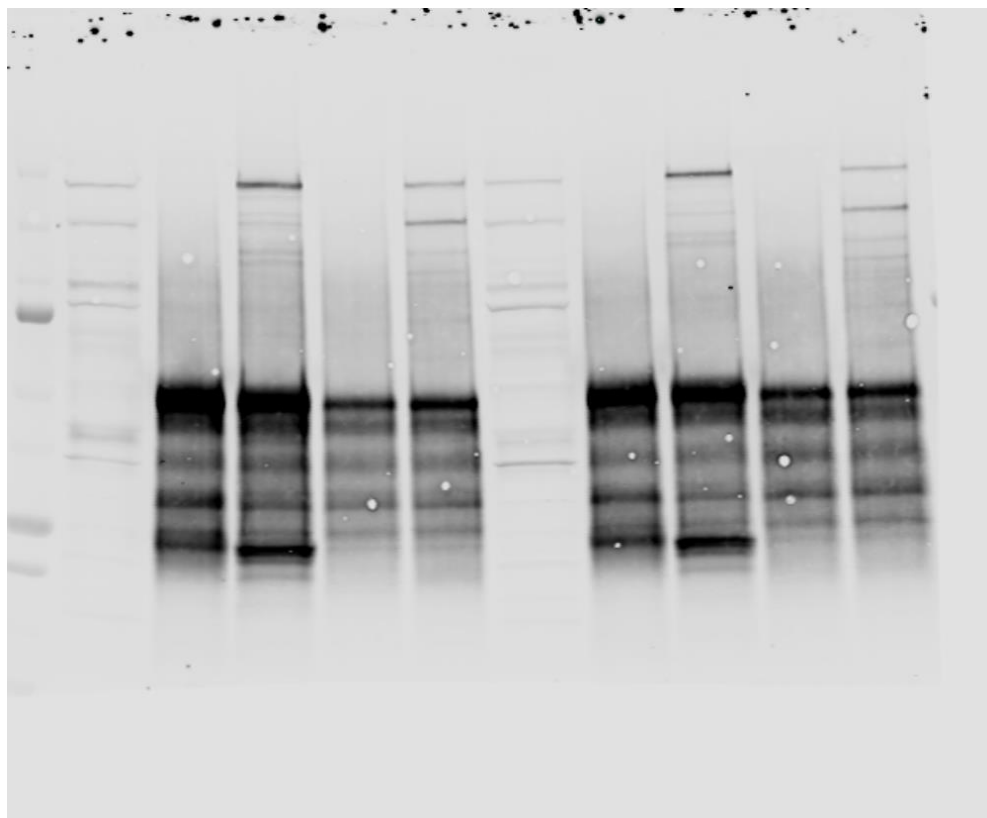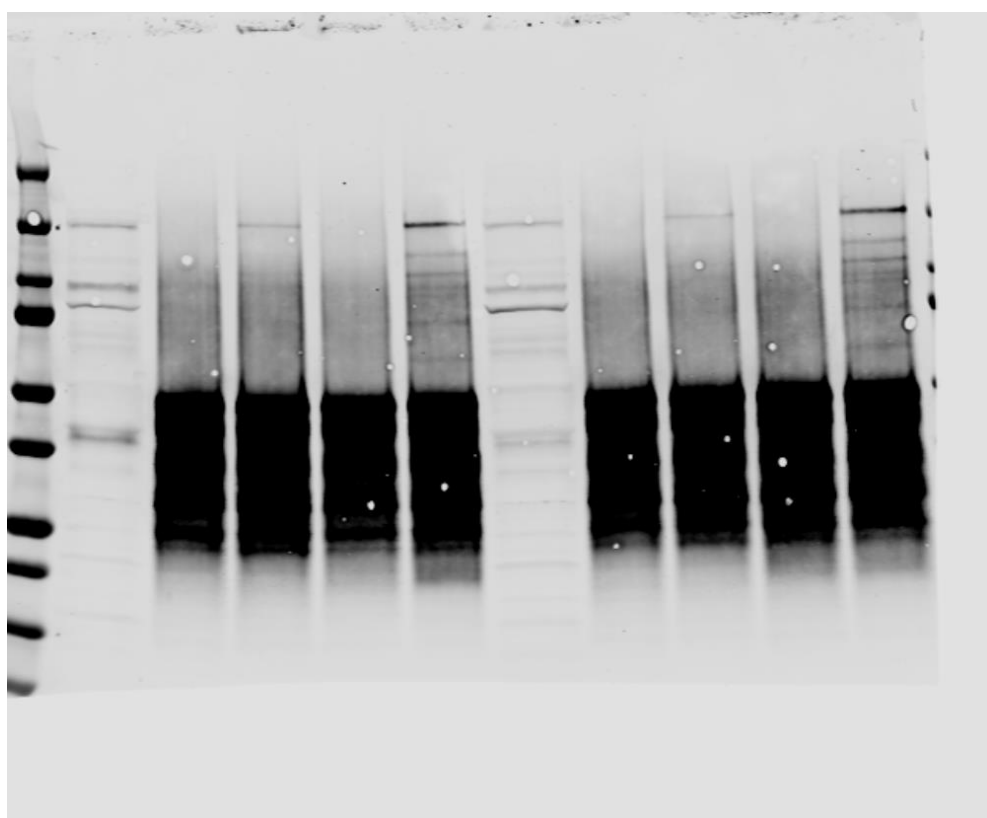

Supplement: Supplementary file 1 [file LSA-2022-01770_SdataF4.pdf]
